# Nutrition, hydration and supplementation considerations for mountaineers in high-altitude conditions: a narrative review

**DOI:** 10.3389/fspor.2024.1435494

**Published:** 2024-11-07

**Authors:** Ewa Karpęcka-Gałka, Barbara Frączek

**Affiliations:** ^1^ Doctoral School of Physical Culture Sciences, University of Physical Education in Krakow, Cracow, Poland; ^2^ Department of Sports Medicine and Human Nutrition, Institute of Biomedical Sciences, University of Physical Education in Krakow, Cracow, Poland

**Keywords:** recommendations, mountaineering, nutrition, diet, mountains, himalaism, hydration, supplementation

## Abstract

Staying and climbing in high mountains (>2,500 m) involves changes in diet due to poor access to fresh food, lack of appetite, food poisoning, environmental conditions and physiological changes. The purpose of this review is to summarize the current knowledge on the principles of nutrition, hydration and supplementation in high-altitude conditions and to propose practical recommendations/solutions based on scientific literature data. Databases such as Pubmed, Scopus, ScienceDirect and Google Scholar were searched to find studies published from 2000 to 2023 considering articles that were randomized, double-blind, placebo-controlled trials, narrative review articles, systematic reviews and meta-analyses. The manuscript provides recommendations for energy supply, dietary macronutrients and micronutrients, hydration, as well as supplementation recommendations and practical tips for mountaineers. In view of the difficulties of being in high mountains and practicing alpine climbing, as described in the review, it is important to increase athletes’ awareness of nutrition and supplementation in order to improve well-being, physical performance and increase the chance of achieving a mountain goal, and to provide the appropriate dietary care necessary to educate mountaineers and personalize recommendations to the needs of the individual.

## 1 Introduction

High altitude climbing is alpinism defined as ascending difficult mountain terrain using appropriate technique and equipment. It is a sport combining rock climbing, ice climbing, ski mountaineering and glacier travel (1). A special variety of climbing is Himalayanism i.e., climbing practiced in the highest mountains in the world, whose peaks exceed 7,000 m the (Himalayan mountain ranges, Karakorum, Daxue Shan, Hindu Kush, Kunlun, Pamir and Tienshan) (2). Interest in high-mountain climbing is growing every year, including among mountaineers at the amateur level. The Himalayan Database describes all known attempts to climb Nepalese and border peaks between 1905 and 2019, where 10,363 expeditions were recorded, involving 60,162 climbers and 28,587 high-altitude porters (3, 4). Table 1 shows the characteristics of altitude ranges referred to in the article (5, 6).

**Table 1:** Definitions of altitude and associated physiological changes (5, 6).

Altitude	Definition	Physiological changes
1,500–2,500 m	Intermediate altitude	Physiological changes detectable. Arterial oxygen saturation >90%. Altitude illness rare but possible with rapid ascent, exercise, and susceptible individual.
2,500–3,500 m	High altitude	Altitude illness common when individuals ascend rapidly.
3,500–5,800 m	Very high altitude	Altitude illness common. Arterial oxygen saturation <90%. Marked hypoxemia during exercise. 5,800 m is altitude of the highest permanent habitation.
>5,800 m	Extreme altitude	Marked hypoxaemia at rest. Progressive deterioration despite maximal acclimatisation. Permanent survival is not thought to be possible.
>8,000 m	“Death zone”	Prolonged acclimatisation (>6 weeks) is essential. Most mountaineers require supplementary oxygen to climb safely. Arterial oxygen saturations about 55%. Rapid deterioration is inevitable and time spent above this altitude is strictly limited.

Staying and climbing in high mountains involves a change in diet due to poor access to fresh food, lack of appetite, food poisoning, harsh environmental conditions and physiological changes (7, 8). According to the definition of altitude above 2,500 m altitude illness common when individuals ascend rapidly (Table 1) (5, 6). Mountaineers have to deal with reduced atmospheric pressure, leading to the development of hypobaric hypoxia, intense solar radiation, heavy rain or snow, strong winds and low temperatures with large diurnal fluctuations, as well as the risk of avalanches and falling rocks (1, 9). Physical performance and aerobic capacity are impaired under hypoxia, and maximal oxygen consumption (VO_2_max) decreases by 6% for every 1,000 m of elevation gain (10). Acclimatization to prolonged hypoxia is regulated at the molecular level by hypoxia-inducible factor (HIF). HIF-induced genetic reprogramming includes increased erythropoiesis, angiogenesis, increased glycolytic enzyme activity and inhibition of oxidative phosphorylation (11, 12). Physical or mental stressors activate the hypothalamic-pituitary-adrenal (HPA) axis, resulting in the release of glucocorticoids that induce the activity of cellular reduction and oxidation systems (13).

Due to the conditions that prevail in the high mountain environment, low supply of antioxidant components with food, heavy physical exertion and mental stress there is an increased production of reactive oxygen species (ROS), which is referred to as oxidative stress (14–19). The effects of ROS include the development of neurodegenerative processes, which are recognized in mountaineers returning from high-altitude expeditions, as well as cognitive impairment and the appearance of pathological changes in brain structures, indicating the development of vasogenic edema (20) and damage to the intestinal barrier, leading to consequences such as bacterial translocation and local/systemic inflammatory reactions (21). Visitors to high mountains often experience gastrointestinal distress and loss of appetite, which can result from hypoxia itself, but can be exacerbated by infection. Acute short-term exposure to altitude appears to increase leptin levels through hypoxia-inducible factor HIF 1*α* and contributes to decreased appetite (22, 23). In addition, several other hormonal markers may contribute to this effect (24). This appears to be largely dependent on the duration of exposure to hypoxia, with the greatest weight loss, due primarily to loss of free fat mass, observed during the first weeks at altitude (8, 25–27), so monitoring energy intake to limit weight loss should be a priority in mountaineers.

Staying in high-altitude conditions poses a risk to mountaineers’ health and life, and requires them to have adequate knowledge, preparation and experience to prevent the negative effects of climbing at high altitude, as well as the ability to make quick decisions and manage the risks of staying in exposed terrain. One mistake can cost the loss of life, hence full focus on the tasks performed during climbing under conditions of increasing fatigue is essential. Adequate nutrition, hydration and appropriately selected supplementation (of micronutrients that are deficient in high-mountain conditions, antioxidants and ingredients that improve exercise performance) help optimize mountain activities and can help prevent nutritional deficiencies that negatively affect health, cognition and physical performance. Developing nutritional and supplementation recommendations requires an understanding of the physiological adaptations to altitude that occur in the human body, as well as the consequences of staying in an unfavorable high-altitude environment, which are weight loss and decreased appetite. The purpose of this review is to summarize current knowledge on nutrition, hydration and supplementation in high-mountain (>2,500 m) conditions. Previously published studies have described recommended solutions for mountaineers in the context of nutrition and hydration. Our review completes this information with data on supplementation in high mountains in order to create a compendium of practical knowledge necessary to develop an optimal nutritional and supplementation strategy to help mountaineers.

## 2 Materials and methods

The review was conducted using the following databases: Pubmed, Scopus, ScienceDirect for studies published in English, from January 2000 to October 2023. Search terms included the following keywords: “mountains”, “mountaineers”, “himalayans”, “hypoxia”, “high altitude”, “nutrition”, “hydration”, “diet”, “energy expenditure”, “macronutrients”, “micronutrients”, “minerals”, “vitamins”, “supplements”, “nitrates”, “beetroot juice”, “N-acetylcysteine”, “Ginko biloba”, “tart cherry”, “glutamine”, “curcumin”, “omega-3 fatty acids”, “carbohydrates”, “proteins”, “fats”, “probiotics”, “prebiotics”, “short-chain fatty acids”, “caffeine” which were combined with Boolean operators (“AND” and “OR”). A preliminary search identified 2,579 articles. Exclusion criteria included studies involving neonates, newborns, high altitude natives or animals, permanent sojourns at altitude and/or disease related hypoxia. An additional Google Scholar search was conducted to identify other additional studies not identified in the above databases. Literature lists of articles and books were also downloaded. The remaining articles were searched manually. Reviewed articles related to the search terms were evaluated based on the titles, abstracts and content of the articles, and in the end, 207 articles were selected. Articles considered were randomized, double-blind, placebo-controlled trials, narrative review articles, systematic reviews and meta-analyses.

## 3 Discussion

### 3.1 Energy supply and energy expenditure in the context of weight loss of mountaineers during high altitude expeditions

Prolonged exposure to high altitudes is associated with an imbalance between energy expenditure and energy intake, resulting in a decrease in body fat stores, and this relationship increases as altitude increases (28). At altitudes above 5,000 meters above sea level, negative energy balance may result from reduced energy intake due to reduced appetite (29) or partly due to intestinal disorders (28, 30, 31). To offset heat loss and maintain proper body temperature, there may be an additional increase in energy expenditure due to involuntary shivering, activated to increase heat production and use fat stores (32). Changes in body composition may not only be based on an adaptive response to hypoxia, but may also depend on genetic factors (33, 34). Reduction of brown adipose tissue during high-altitude sojourns does not appear to be desirable due to its thermoregulatory functions (maintenance of body temperature) (35, 36) and resulting impairment of physical performance (37). Natural stressors, such as cold, physical exercise, leptin expression and interleukin-6 expression, initiate adipocyte browning towards beige fat, triggering a higher thermogenesis (38). Especially in long-term altitude sojourns, this might be a factor which strongly increases energy expenditure. Both increased leptin expression and increased energy expenditure decrease with the degree of altitude acclimatization and presumably subside after complete acclimatization. This initial period during high-altitude sojourns is characterized by greater consumption of fatty acids recruited from the body's reserves stored in adipocytes (37).

Increased ventilation, due to being in a cold and dry high altitude environment, can also contribute to increased water loss (i.e., non-perceptible water loss), also leading to weight loss (27). Mountaineers lose at least 3% of their body weight after eight days at 4,300 m above sea level and 15% after three months at 5,300–8,000 m above sea level (29). In women, altitude-induced weight changes are smaller compared to men. In a study involving 12 women who climbed to an altitude of 5,050 m above sea level and stayed there for 21 days, no changes were observed in body weight and lean mass compared to baseline values (39).

Negative energy balance leading to depletion of glycogen and fat stores is the main cause of muscle protein catabolism (40). Above 5,000 m above sea level, 60%–70% of weight loss is lean body mass (41, 42). Its loss negatively affects aerobic capacity (43), muscle strength (44), and immune function (45), which can increase the risk of disease and injury in these extreme conditions. At altitudes above 8,000 m, the total number of muscle fibers decreases by about 17% (46, 47). The mechanisms of muscle atrophy are largely unknown, but can be attributed in part to malnutrition and reduced exercise intensity, as well as loss of appetite and impaired digestion or absorption (48, 49). On the other hand, the breakdown of muscle tissue may be beneficial as an adaptation under high-altitude conditions because it increases the density of capillaries in relation to the muscle cell (50). Being in hypobaric hypoxia decreases leucine turnover and uptake from muscle cells (51), which results in the arrest of protein synthesis and is associated with a decrease in mammalian target of rapamycin (mTOR) and is not dependent on food intake (52). This suggests that proper nutrition can offset the loss of muscle mass, but it is not possible to completely prevent muscle atrophy when climbing at high altitudes over time (22).

Analyses using the double-labeled water method in mountaineers ascending Mount Everest indicate that energy expenditure is 1.85–3.0 times that at sea level (53). The energy requirements of mountaineers while climbing in the Himalayas were 4,634 ± 287 kcal/day (5,900–8,046 m) (54), 5,394 ± 1,565 kcal/day (5,300–8,848 m) (42) and 3,248 ± 407 kcal/day (5,300–8,872 m) (53), while the energy supply was, respectively, 3,296 ± 478 kcal/day (54), 2,928 ± 968 kcal/day (42) and 1,791 ± 358 kcal/day (53). In the analyzed studies conducted in the Alps (2,400–3,800 m), the energy requirements of professional climbers from the French Military Group during an ultraendurance alpine climbing race were 10,413 ± 287 kcal/day, and energy supply 3,530 ± 165 kcal/day (55), while in the Cascade Mountains (2,500–3,100 m) involving soldiers during military field training, 4,037 ± 387 kcal/day and 2,357 ± 860 kcal/day, respectively (56). In our own research among Polish mountaineers during an expedition to Pakistan in the Shuijerab Mountain Group and Peru in the Cordillera Blanca (4,000–6,000 m), energy requirements were 4,560 ± 425 kcal/day and energy supply was 2,777 ± 878 kcal/day (57). In a study by Miller et al. the energy requirement of mountaineers summiting a Pangaea Peak in Karakoram Mountains (2,230-6,170 m) was 4,173 ± 848 kcal/day, but energy supply was not determined (58). The majority of studies used the gold standard technique of energy expenditure measurement, the doubly labeled water method (42, 53, 54, 56). An alternative method used by many authors to estimate total daily energy expenditure during mountaineering activities is heart rate recording (55, 57, 59).

A study by Pulfrey and Jones found that mountain climbers staying at extreme altitudes (5,900–8,046 m) covered 70% of their daily energy requirements with diet (54). The results of Watts et al. study conducted in the Cascade Mountains (1,636–3,266 m) showed that technical ice climbing was characterized by generating the highest energy expenditure of all mountaineering activities studied (59). A trail hike with a load averaging 45% of body weight led to an energy expenditure of 7.5 ± 1.9 kcal/min in 2.2 h, with an average peak of 13.4 ± 3.0 kcal/min. On the other hand, technical ice climbing consisting of moving up and down 12 meters in an average time of 14.3 ± 2.6 min required an average energy expenditure of 9.5 ± 3.4 kcal/min, and the peak average value was 14.6 ± 3.5 kcal/min (59). Energy expenditure of experienced climbers during mountaineering was lower than that of inexperienced climbers (59). It was also shown that the estimated energy expenditure increases with increasing slope angle by additional 1.5–2.0 kcal/min for overhangs at 80°–90° and additional 5 kcal/min for overhangs at 102° (60). According to the recommendations and the level of physical activity, the energy requirements of athletes can range from 40 to 70 kcal/kg of body weight/day (kg of b.w.) (2,000–7,000 kcal/day for a 50–100 kg athlete) (61).

Many of the factors influencing weight loss during a high-altitude expedition are beyond the mountaineers’ control (loss of appetite, frequent gastrointestinal distress, increased energy expenditure due to increased thermogenesis and physiological processes determined by being in hypoxia, water loss due to constricted ventilation, loss of lean body mass). The only factor that mountaineers have a real influence on is energy supply. It is therefore worth aiming to increase energy supply to cover energy requirements. This is an important element of an expedition to plan, especially during the acclimatisation phase and in difficult technical terrain (ice climbing), when energy expenditure is higher.

### 3.2 Recommendations for macronutrient dietary supply while at high altitude

Staying in high-altitude conditions alters substrate oxidation for a given level of exercise intensity (62, 63). Modification in energy substrate utilization at high altitude toward carbohydrates (CHO) (64) influences increased dietary carbohydrate requirements for muscle glycogen storage and replenishment, maintenance of body mass, avoidance of hypoglycemia, adequate recovery and optimal glycogen resynthesis (65, 66). A higher intake of CHO is important at every stage of trekking and climbing in high mountains and has a beneficial effect on performance (67, 68). Consuming enough carbohydrates is especially important when there is cold stress and chills (69). Heat can be produced by shivering, which is an unconscious mechanism activated by the central nervous system, triggered by a decrease in body temperature (70). Increased muscle contractions during shivering result in a 2.5-fold increase in energy expenditure, most of which is due to increased carbohydrate oxidation. Cold exposure also results in increased muscle glycogen utilization, resulting from increased plasma catecholamine concentrations (71).

According to dietary recommendations (72), carbohydrate supply during low-intensity and technical exercise, should be 3-5 g/kg of b.w./day. Athletes doing moderate amounts of high-intensity training (about 1 h/day) should provide 5–7 g carbohydrates/kg of b.w./day, while endurance trainers doing moderate to high-intensity training (about 1–3 h/day) should take in 6–10 g carbohydrates/kg of b.w./day. Extreme endurance training (about 4–5 h/day) of moderate to high intensity requires 8–12 g of carbohydrates/kg of b.w./day. In order to improve glycogen resynthesis, it is recommended to consume 1–1.2 g of carbohydrates/kg of b.w. in the first 4–6 h after the end of activity.

Carbohydrate supply should be adjusted not only to the intensity of the exercise, but also to the stage of the expedition. During base camp at low physical activity, this supply should be about 3–5 g CHO/kg of b.w./day, while during trekking and/or climbing a minimum of 6 g/kg of b.w./day. According to the study by Friedlander et al. (73), carbohydrate intake in people at high altitude should be at least 60% (6–8 g/kg b.w./day) of total energy intake. These guidelines are based on studies showing that total carbohydrate oxidation during high-altitude exercise is greater than during exercise of the same intensity at sea level (74) leading to faster utilization of the body's carbohydrate stores (i.e., muscle and liver glycogen and blood glucose). A high-carbohydrate, low-fat diet at altitude increases the respiratory quotient (RQ). If only fats are used for energy production, this coefficient is 0.7, while using carbohydrates (or proteins) increases to a value close to 1. The effect of this change in RQ is to increase the partial pressure of oxygen, resulting in an increase in arterial blood oxygen saturation (75–77). In addition, at high altitudes, carbohydrate oxidation was considered the preferred metabolic pathway for aerobic exercise because it provides the highest adenosine triphosphate (ATP) yield per mole of oxygen (78). The study by McClelland et al. shows that carbohydrate utilization does not change after altitude acclimatization and that metabolic fuel utilization is mainly influenced by relative exercise intensity, as at sea level (79). In women, unlike men, carbohydrate utilization decreased while at 4,300 m above sea level (80). McKay et al. (81) showed in a study that short-term CHO restriction increased concentrations of the iron-regulating hormone, hepcidin, thereby potentially reducing post-exercise iron absorption. These studies were not conducted in mountainous terrain, so it would be appropriate to conduct an analogous study among mountaineers to assess the impact of a low-carbohydrate diet on the athletes’ health. Analysis of the carbohydrate supply in the diet of mountaineers during an expedition in the Himalayas indicates either too low [39.5% of the energy supplied (82)] or the right amount [52.7% (57), 52% (83), 51.8% and 67.7% (84)].

From a practical point of view, environmental conditions in high mountains should be taken into account, especially low temperatures, which may prevent the consumption of carbohydrate snacks such as bars due to their freezing. Based on our experience, we recommend that snacks taken to the mountains are first tested at low temperatures. While food preparation is not a problem in base camp conditions (it is possible to take more time to cook and consume the meal unhurriedly, relying on valuable sources of complex carbohydrates), during a summit attack, digestive comfort and ease of meal consumption is a primary requirement. Snacks must also be convenient to eat with warm gloves.

Mountaineers practicing high altitude climbing should provide adequate amounts of protein to prevent weight loss and ensure adequate amounts of this nutrient to build and repair tissues (85). An adequate supply of protein, especially branched-chain amino acids, is key to regulating muscle protein synthesis (86). International sports organizations recommend that the protein intake of physically active individuals should be between 1.2 and 2.0 g/kg of b.w./day, as recommended by the Academy of Nutrition and Dietetics, Dietitians of Canada and the American College of Sports Medicine (ACSM) (72), which is confirmed by the guidelines of The International Society for Sports Nutrition (ISSN), which give a range of 1.4–2.2 g/kg of b.w./day (61). The protein supply of mountaineers’ diets should be adapted to the stage of the expedition and the intensity of the effort. During an energy deficit, it is recommended to increase the protein content of the diet up to 1.6 g/kg of b.w./day, which improves nitrogen balance and maintains lean body mass (87). The protective effect of higher protein supply on muscle and whole-body protein homeostasis is impaired when the energy deficit is more than 40% of daily energy requirements. It was found that a high-protein diet (2 g protein/kg of b.w./day) did not spare lean body mass in military personnel at an altitude of 4,300 m during 21 days of energy deficit. The doubling of protein intake (compared to the standard diet group) resulted in a parallel increase in protein oxidation, suggesting that a greater proportion of dietary amino acids were then oxidized for energy production. Consequently, the availability of amino acids to support protein balance was limited (88). In studies analyzing the nutrient content of mountaineers’ diets in high-altitude conditions, the protein supply was most often too low in relation to recommendations for athletes i.e., 1.1 g/kg of b.w./day (57), 1.2 and 1.3 g/kg of b.w./day (84) or an appropriate 1.5–2.5 g/kg of b.w./day (82). In view of the endurance exercise undertaken by mountaineers, the dietary protein supply should be a minimum of 1.4 g/kg of b.w./day, taking into account high-quality protein and distributing it to the main meals and snacks during the climb or approach.

Foods rich in fat are high in calories, which in the mountains can help prevent weight loss. According to recommendations, athletes should consume a moderate amount of fats from 20% to 35% of their daily caloric needs, while during regular high-volume training they can safely take in up to 50% (61, 72, 89). A high-fat diet appears to be beneficial during high-altitude sojourns (37), due to increased leptin expression, resulting in decreased appetite and increased β-oxidation of fatty acids. It is worthwhile for mountaineers to also be guided by their appetite and select snacks in the mountains according to their own preferences and tolerance of their digestive system. In the Karl et al. study, mountain climbers preferred high-fat foods after weight loss and acclimatization (90). Prolonged exposure to altitude reduces the consumption of free fatty acids during physical activity (62). In the studies conducted so far that analyzed the nutrient content of mountaineers’ diets, the fat content was too high in relation to the recommendations for athletes i.e., 45.5% (82), 38% (84), 36.8% (57), 36.1% (83), and only the Italian group described in the study by Bondi et al. (84) provided relatively little fat (24%). From our perspective mountaineers staying in high-mountain conditions should rely on valuable sources of fat, including in their diet products rich in poly- and monounsaturated fatty acids, such as vegetable oils (olive oil, flaxseed oil), nuts, seeds. Mountaineers choosing products in the mountain diet should be guided primarily by appetite and adjust the supply of this component to the individual tolerance of the digestive system.

### 3.3 Prevention of micronutrient deficiencies in mountaineers’ diets

Due to limited access to fresh vegetables, fruits and many protein-containing foods, the supply of minerals and vitamins in mountaineers’ diets may be too low. To our knowledge, there is a lack of research indicating what the micronutrient requirements of the diet are in high-altitude conditions, and studies conducted so far emphasize the role of iron, B vitamins, antioxidant vitamins and minerals, and vitamin D. Further research is needed to formulate recommendations for the supply of micronutrients in high altitude conditions.

Given the importance of haemoglobin levels for aerobic power, optimal iron content is particularly important for endurance athletes. Furthermore, they are at risk of increased iron loss from sweat, urine, through the gastrointestinal tract and due to haemolysis and exercise-related blood loss, through injury and menstruation (91). At high altitudes, iron requirements are increased due to erythropoiesis (92). This process may be impaired in athletes with low iron levels (93). Adequate iron stores are required to sustain the hypoxia-induced increase in heme synthesis and iron-dependent enzyme production during prolonged exposure at altitude (94). Iron intake was insufficient during the stay in the Alps (3,200–3,616 m) and was 10.4 ± 1.73 mg/day (83) and 14 ± 4 mg/day during an expedition in the Himalayas (1,070–5,143 m) in the Nepalese group and 24 ± 3 mg/day in the Italian group (84). According to the recommendations of the International Olympic Committee, 8–10 weeks before the start of training activities above 2000m, ferritin levels should be assessed (95). It is suggested to increase iron intake or oral supplementation when serum ferritin levels are less than 30 nmol/L in women and 40 nmol/L in men (95), however, these pre-altitude ferritin cut-offs, in combination with iron supplementation, have not been scientifically validated. According to Stellingwerff et al. (67) it is recommended to perform pre-altitude blood tests about 4–6 weeks prior to allow for a more precise assessment of ferritin levels, so that appropriate oral supplementation can be started about 2 weeks before and during altitude exposure, if necessary. Recommendations for iron supplementation before mountain activity were established on ferritin cut-offs of <100, ∼100–130 and >130 ng/ml and are based on interpolation and/or extrapolation of existing data (94, 96–98). Current findings suggest that most athletes in hypoxic environments increase hemoglobin mass by taking ∼100–200 mg of elemental iron per day orally, with most of the evidence related to iron salts. We recommend involving a sports medicine physician in the process of assessing the need for iron supplementation prior to high-altitude exposure and determining its dose in the event of iron deficiency or low ferritin concentrations. Excessive iron supplementation and clinically elevated endogenous iron stores can have negative health consequences (67, 99). Ferritin limits need further scientific validation, as there is currently a lack of studies evaluating the body's response to iron intake in mountaineers staying above 3,000 m above sea level. It is important for mountaineers to include rich sources of iron (meat, fish, pulses, whole grains, dairy products) in their diet before and during the expedition. When consuming iron-containing products, it is also important to consider its bioavailability. Heme iron (from meat) has a higher absorption capacity (∼5%–35%) than non-heme sources (∼2%–20%) (100). The presence of vitamin C and meat, poultry and fish can increase the absorption of nonheme iron, while substances such as polyphenols, phytates or calcium that are part of tea, coffee, whole grains, legumes and dairy products can decrease the amount of nonheme iron absorbed from a given meal (101). In addition, it is worth considering a probiotic (102, 103) and lactoferrin (104–106) to improve iron bioavailability. Lactoferrin increases intestinal iron absorption by binding to iron and improves hemoglobin and serum iron levels, thus maintaining iron homeostasis in the body and cells (106). Optimal levels of vitamin D (30–50 ng/ml) and B_12_ (400–700 pg/ml) can probably help improve iron status and thus avoid anemia (107–109).

Hypobaric hypoxia leads to reduced oxygen delivery to the brain, and can subsequently contribute to cognitive impairment and increased risk of dementia, including Alzheimer's disease (110). High altitude cerebral edema and hypoxia cause cognitive impairment and dementia associated with white matter pathology (111). Whether exposure to hypoxia at high altitudes causes irreversible brain damage is debatable. In an magnetic resonance imaging (MRI) study of 35 mountaineers, the authors concluded that there is sufficient evidence of brain damage after high altitude climbing, and that amateur climbers are at higher risk of brain damage than professional climbers (112). In the study, Garrido et al. showed that Sherpas highlanders have better brain protection when exposed to extreme altitude compared to a group of elite lowland mountaineers who had climbed more than 8,000 m, and a control group (113). A study by Tunali et al. describes the case of a 57-year-old man who developed symptoms of acute mountain sickness after climbing 5,416 m in Nepal. Several months after descending the mountain, he developed symptoms such as loss of empathy, impaired speech, problems with perceiving and expressing emotions, and an increased interest in sugary foods. The patient's MRI and PET/CT results showed frontotemporal neurodegeneration (111). Metabolic functions of B vitamins and their role in neurochemical synthesis can be seen as having a particular impact on brain function (114). Folic acid, vitamin B_12_ and iron play a key role in erythropoiesis (115). Krzywanski et al. recommended that vitamin B12 serum concentrations in athletes training at sea level should be maintained between 400 and 700 pg/ml to support haemoglobin synthesis and improve red blood cell markers, with regular monitoring to consider supplementation when vitamin B12 levels are <400 pg/ml (108). However, there is a lack of studies assessing the optimum serum concentration of B vitamins and the requirement for these vitamins in high-altitude conditions. Currently, there is insufficient evidence to recommend supplementation of B vitamins in high-altitude conditions, given their role in erythropoiesis and to improve cognitive functions.

Vitamin D plays an important role in immune, muscle and cardiovascular function, inflammatory response, protein synthesis, cell growth and regulation of the musculoskeletal system (116–118). Many athletes have insufficient levels of vitamin D, and most often serum levels are below 20 ng/ml, especially during the winter months (119). According to Zhang et al. (120), to achieve optimal anti-inflammatory effects of vitamin D, it is important to maintain serum vitamin D levels >30 ng/ml. According to current recommendations, in normal-weight adults, cholecalciferol or calcifediol supplementation should be implemented and continued under supervision of a medical doctor until an optimal serum concentration of >30–50 ng/ml of 25-hydroxyvitamin D (25(OH)D) is achieved and maintained (116). Kasprowicz et al. (109) have shown that high doses of vitamin D (10,000 IU/day) can prevent the decline in serum iron levels after exercise. Kasprzak et al. (83) have observed a decrease in 25(OH)D serum concentrations among mountaineers during a 14-day stay at 3,200-3,616 m above sea level, associated with modulation of immune processes. This is consistent with a study conducted at an altitude of 5,400-6,700 m, for about 4 months, involving 221 male volunteers serving in the Indian Army (121), in whom a decrease in serum 25(OH)D concentrations was associated with skeletal deterioration at extreme altitudes (121). Mountaineers staying in high-altitude conditions with low temperatures and UV radiation, need to protect their skin from burns, and additionally use down suits, both of which prevent skin synthesis of vitamin D. Given how important the role of vitamin D is in the context of the immune system and the fact that its deficiency is associated with a higher risk of anemia (107), it is important to monitor the concentration of this vitamin before the expedition, adjust the appropriate dose to the current serum concentration, and continue supplementation during the expedition.

Staying at high altitudes, associated with reduced oxygen pressure, can result in increased production of reactive oxygen and nitrogen species and the development of oxidative stress (15). Excessive production of RONS, which exceeds endogenous antioxidant defenses, can lead to damage to lipids, proteins and DNA, thereby impairing cellular and immune function, causing delayed post-workout recovery (122). The supply of antioxidant-rich dietary components can be a helpful intervention in the fight against altitude-induced oxidative stress. It seems most prudent to include a large amount of antioxidant-rich foods, including vitamin C, beta-carotene, vitamin A, vitamin E, selenium and zinc in the daily diet of mountaineers. Freeze-dried fruits, vegetables and their powdered forms can be valuable sources of antioxidant vitamins in high-altitude conditions. An intervention at 2,320 meters above sea level to increase the intake of antioxidant-rich foods, served as snacks between meals, not only increased the intake of antioxidants, but also improved the overall amount of macro- and micronutrients in the athletes’ diets (123). It would be advisable to test such a strategy in the higher parts of the mountains as well.

### 3.4 Importance of hydration and electrolyte supply during high altitude sojourn

Hypoxia occurring in high-mountain conditions increases fluid loss through hyperventilation and contributes to dehydration, which reduces aerobic capacity (124). Dehydration causes changes in electrolyte concentrations in the body, resulting in stress on the cardiovascular system as plasma volume decreases. Impaired cardiovascular function reduces cutaneous blood flow and the body's ability to dissipate heat to the environment (125). In addition to these physiological effects, dehydration can impair cognitive function and concentration (126).

Dehydration has not been shown to increase the risk of acute mountain sickness (AMS) (124), although control of fluid output is thought to be important in preventing the onset of AMS, as clinically, measures to prevent excessive fluid retention are likely to reduce the symptoms of AMS (127). Fluid requirements in high-altitude conditions are higher than at sea level also due to hyperventilation, low humidity, sweat losses (127, 128) and increased diuresis as a result of down-regulation of the renin-angiotensin-aldosterone system, especially during the first days of the stay (129). Optimal hydration may also be limited by the time and fuel resources needed to prepare potable water, sourced from the glacier (130). Another factor that contributes to dehydration is reduced thirst at high altitudes (131).

Urine colour analysis is a simple and convenient method of determining the whole-body hydration status (132, 133). Numerous studies using the subjective eight-point colour scale have shown that an increase in dehydration of the subject results in a darker urine colour (134). Fluid supply should be responsive to thirst and sufficient to prevent dehydration and avoid infrequent dark-coloured urination. Attempting to take in large amounts of fluids in a short period of time should be avoided (75). At 4,300 m water loss from the respiratory tract can be increased to 1,900 ml/day in men (85) and 850 ml/day in women (135), and urinary excretion by 500 ml/day (136). Water loss in mountaineers of Mount Everest (5,000–8,872 m) was 3.3 ± 0.6 L/day (53), while in people with low physical activity, engaged in cooking, melting snow, repairing tents, stationed at 6,542 m for 21 days, 3.0 ± 0.5 L/day (137). Another study analyzed water balance at sea level and at an altitude of 4,350 meters. Water loss decreased from ∼4.5 to 3.5 L/day, mainly as a result of a decrease in ambient temperature of ∼10°C (138). There was also a study among mountaineers who climbed Denali in Alaska. Based on two methods of measuring hydration status (urine specific gravity and ultrasound measurements of inferior vena cava size and IVC-CI collapse index), it was found that about half of the mountaineers in the study were dehydrated (130). In contrast, in a survey conducted by Karpęcka-Gałka et al. (57), almost half of mountain climbers drank 2-3 L of fluids per day in the mountains, while the rest of the respondents took even smaller amounts of fluids per day, which can lead to dehydration. Average water supply (per person per day) was monitored in a study conducted by Bondi et al. (84) in a group of Italians and Nepalese during an expedition in the Himalayas (1,070–5,143 m), reaching 3,099 ± 462 g/day and 3,240 ± 310 g/day, respectively.

Mountaineers in high-mountain conditions use glacier water as a source of hydration and for cooking, characterized by low mineral content, so it is worth considering electrolyte supplementation. An additional complication that arises in high mountains is the reduced boiling point, as a result of which prepared meals must take longer to cook (139). In the absence of water treatment tablets, there is a high risk of parasite infection. *Giardia lamblia*, an intestinal parasite that causes diarrhea, is found in high-altitude regions (140). It should be emphasized that recommendations for fluid replenishment at high altitude should be individualized due to different rates of fluid and electrolyte loss, urine color control and symptoms of AMS, which causes water retention in the body. According to ACSM expert recommendations, the daily fluid requirement is 4–5 L during altitude training and competition (72), while ISSN encourages individual monitoring of hydration status to determine athletes’ fluid requirements (61). General recommendations during mountain activities include intake of 400–800 ml/h fluids with 0.5–1 g Na/L water (139).

### 3.5 Recommendations on the use of dietary supplements during high altitude expeditions

Due to poor access to food products at high and extreme altitudes, supplementation may be the only solution to provide adequate energy and essential nutrients, as well as to improve the functioning of the body in harsh environmental conditions. To date, there have been many studies confirming the effects of ergogenic agents during physical activity (141), however, there are a limited number of reports on supplementation in hypoxic or high-altitude conditions. Supplements with potential beneficial effects on exercise capacity, sleep quality, the immune system, the intestinal barrier, the gut microbiome and more in high-altitude conditions are described below. However, most of them require further research.

#### 3.5.1 Carbohydrate supplements

Carbohydrate supplementation during exercise by non-acclimatized men has improved performance in a test conducted after 3 days of exposure at an altitude of 4,300 m, with a simultaneous 30% deficit in delivered energy applied (68). In a study involving men acclimated to altitude and in a state of energy balance, there was no benefit of carbohydrate supplementation on time-trial performance during the first and third days of altitude exposure (142). From the above studies, despite many differences in methodology, it can be concluded that the potential ergogenic effect of supplemented carbohydrates on exercise capacity may be modulated by acclimatization. In a recent study evaluating the effect of carbohydrate supplementation on aerobic exercise performance in non-acclimated men after 5 h of exposure to an altitude of 4,300 m and after 22 days of acclimatization with an accompanying 40% energy deficit, there was no improvement in aerobic capacity (143). Based on the results of the study by Bradbury et al. (143), it is recommended that people planning to stay or engage in physical activity (e.g., alpine climbing) in high mountains prioritize carbohydrate intake while still at sea level in order to optimize glycogen stores before going into the mountains.

Although carbohydrate requirements to meet energy expenditure at high altitude increase, there is a paradoxical impairment in the ability to utilize exogenous carbohydrates for, at least, the first day after ascent. The inability to oxidize and functionally benefit from exogenous carbohydrate intake during high-altitude activity coincides with hyperinsulinemia, accelerated glycogenolysis and reduced peripheral glucose uptake. These responses are consistent with hypoxia-mediated deregulation of metabolism, reflecting insulin resistance. The findings also suggest a role for the gut microbiome in host metabolism, bioenergetics and physiological responses at high altitude, suggesting that the gut microbiome is a potential mediator of hypoxia-mediated metabolic deregulation (144). Pasiakos et al. (144) have indicated a lack of ergogenic effects of carbohydrate supplementation at altitude, which questions the effectiveness of recommending carbohydrate supplementation to maintain physical performance after ascending to high altitude. Thus, more research is needed to develop appropriate carbohydrate supply strategies that take into account the effects of hypoxia on insulin sensitivity and substrate oxidation to optimize the activities performed in high-altitude conditions. From our perspective, given the challenging conditions during high-mountain climbing, it is worthwhile for mountaineers to consider carbohydrate-supplying supplements (energy gels, gummies, carbohydrate jellies, carbohydrate bars, isotonic drinks) that are low in weight and easy to open at low temperatures in order to increase dietary carbohydrate supply, as well as to increase energy intake.

#### 3.5.2 Protein supplements

If the protein supply does not cover the body's need for this nutrient, supplementation with a protein can be considered. Stimulation of muscle protein synthesis and/or reduction of proteolysis with a low-volume protein supplement rich in branched-chain amino acids, especially leucine, seems to be the best strategy to protect the body of a high altitude climber from an undesirable decrease in lean body mass (22). However, the results of studies conducted so far have not shown a benefit in this aspect when mountaineers supplemented their diet with a preparation of branched-chain amino acids (145, 146). Protein increases feelings of satiety (147), which is not a beneficial effect at high altitude, where mountaineers struggle with altitude anorexia (148). There is still a lack of studies evaluating the effect of protein supplementation on feelings of satiety at high altitude. Based on our experience, a diet rich in protein may not meet the expectations and dietary preferences of high altitude climbers, so when staying in high mountains above base camp, it is worth considering supplementation with a protein supplement to cover the daily requirement of this component, which also gains justification given the lower availability of food products that are sources of protein.

#### 3.5.3 Antioxidants

Exogenous antioxidants neutralize free radicals, so it would seem logical that supplementation with these compounds could be a helpful intervention against altitude-induced antioxidant stress. While some studies have shown that antioxidant supplements have a modulating effect on oxidative stress and AMS symptoms at high altitude (149), more recent studies indicate that there is no such effect (150). After all, supplementation with antioxidants, especially in the early phase of the altitude exposure, can be counterproductive and potentially weaken or delay acclimatization to altitude (151). On the other hand, the results of the study of Koivisto et al. indicate that increasing the intake of antioxidant-rich foods during altitude exposure caused the expected increase in antioxidant capacity and attenuated some altitude-induced systemic inflammatory biomarkers in athletes (152). Undeniably, pro-oxidant-antioxidant balance is paramount for altitude acclimatization, but the question of planning an appropriate antioxidant-related nutritional and supplementation strategy remains unclear.

#### 3.5.4 Caffeine

Caffeine is a supplement with a very well-documented effect, used to improve exercise capacity, neuromuscular function, alertness and concentration, as well as reduce perceived fatigue during exercise (153). The benefits of its supplementation are observed in sports of an endurance nature, requiring dexterity and precision, as well as short-duration, supramaximal or repeated high-intensity efforts (141). Mountaineering is an endurance sport with elements of intensive effort of a strength nature (55). Thus, it is worth considering caffeine supplementation among mountaineers, given the nature of the sport and the extreme fatigue and impaired concentration resulting from the harsh high-altitude conditions. Caffeine supplementation can also have a positive effect on cognitive function, and according to a study by Zhang et al. (154), low doses of caffeine (3 mg/kg of b.w.) have better effects in terms of improving cognitive function and brain activation. In a study conducted by Tian et al. (155), caffeine alleviated high altitude pulmonary edema (HAPE) and reduced oxidative stress and stabilized mitochondrial morphology in alveolar type 1 cells under hypoxic conditions. The results of the study presented here suggest that caffeine may be a potential treatment for abnormal acclimatization resulting in HAPE (155). Due to the need for mountaineers to remain focused despite extreme exhaustion throughout the expedition, caffeine could be used in high-altitude conditions. It is worth considering caffeine supplementation about 60 min before situations requiring sharpened attention (blood caffeine concentrations peak at this time) (156) such as technically difficult sections of the route, before rappelling after the climb or on the way back from the summit. However, further research is needed to confirm this hypothesis.

#### 3.5.5 Beetroot juice

Beetroot juice (the nitrates it contains) is a supplement that has attracted a lot of interest in recent years for its effects in improving the ability to perform long-term moderate-intensity exercise and repeated high-intensity short-term exercise. The mechanism of action of nitric oxide (NO) is based on improved blood flow through blood vessels, lower ATP consumption during force generation by muscles, reduced aerobic cost of effort, and increased biogenesis and mitochondrial performance (141, 157–159), which can potentially improve athletic performance. It is recommended to consume a dose of 6–8 mmol (∼350–500 mg) of nitrates 2–3 h before exercise. Another possible strategy is to consume 6–8 mmol of nitrates per day for several days prior to competition (160).

There are studies confirming increased exercise economy by about 5%–10% in hypoxic situations and/or better performance in recreational athletes after nitrate supplementation (161–163). In high-performance endurance athletes in hypoxic conditions, beetroot juice supplementation was not beneficial (164–167), but there is also a study supporting its effectiveness (168). Current evidence suggests potential improvement in endothelial function at high altitude after nitrate supplementation (169). In another study carried out in hypoxia assessed that the nitrates in beet juice do not prevent altitude sickness and should not be recommended as a preventive or ergogenic measure for already existing altitude sickness (170). Hannis et al. (171) in a study conducted with young men while trekking to Everest Base Camp showed that oral nitrate supplementation using beetroot juice was safe and feasible at altitude, and that dietary nitrate did not change mountain sickness symptoms or alter physiology. These results are consistent with a study performed by Cumpstey et al. (172) in the Alps (4,559 m), in which dietary nitrate supplementation was shown to be well tolerated at high altitude and to significantly increase lung NO availability and NO metabolite concentrations in saliva and exhaled breath condensate, and was not associated with changes in haemodynamics, oxygen saturation or the development of AMS. Similarly, a study conducted by Marshall et al. (173) with the British Military during a trek in the Himalayas (1,400–5,755 m) confirmed that dietary nitrate supplementation may ameliorate the decline in physical performance associated with staying at high altitude and has no effect on the incidence of high altitude illness. In the study by Bakker et al. (174), acute dietary nitrate ingestion offset the decline in endothelial function in healthy young participants during an expedition in Nepal (28 days >2,500 m, including a peak of 3,700 m).

Most of the published studies have examined the effects of nitrate supplementation at simulated altitude (161–168), which is often considered an incomplete substitute for “real” altitude. There is ongoing debate about potential differences in the physiological response to normobaric hypoxia and terrestrial altitude (175), including potential disparities in NO metabolism (176). The research conducted so far at terrestrial altitude is promising (171–174). Further studies are needed to evaluate the effects of nitrate−at terrestrial altitude and/or contrasting the effects of nitrate−supplementation between terrestrial and simulated altitude.

#### 3.5.6 Prebiotics and probiotics

Gastrointestinal complaints are often reported during climbing at high altitudes (>2,500 m), although their etiology is unknown. Hypoxia and oxidative stress can damage the intestinal barrier, consequently leading to bacterial translocation and local/systemic inflammatory reactions. Preliminary data suggest that prolonged exposure to hypoxia can damage the intestinal barrier through changes in immune function, microbiota or mucosal layers. Exercise may worsen the intestinal damage associated with high altitude through additional reductions in visceral circulation and greater hypoxemia (21). Kleessen et al. analyzed fecal samples and serum from 7 mountaineers participating in a 47-day expedition to the Himalayas. The results of the study indicate a change in the composition of the gut microbiota after exposure to very high altitudes (>5,000 m). The number of bifidobacteria and species belonging to the *Atopobium*, *Coriobacterium* and *Eggerthella lenta* groups decreased, while the number of potential pathogenic bacteria from the gamma Proteobacteria subgroup and specific *Enterobacteriaceae*, such as *Escherichia coli*, increased. Changes in the composition of the microbiota may be associated with changes in indicators of the immune system and result in the deterioration of mountaineers’ health (177). Karl et al. (178) have observed increased intestinal permeability in a group of healthy, physically active but non-acclimated men after a rapid (transported by airplane and car), 22-day exposure to an altitude of 4,300 m. The findings are consistent with Dinmore et al. (179); however, they should be interpreted with caution due to the limitations of the ongoing projects.

Acetate, propionate and butyrate, short-chain fatty acids (SCFAs) that are produced by some gut microbes mainly during the fermentation of dietary fiber, are key mediators of intestinal permeability and may protect the intestinal barrier from hypoxia-induced dysfunction (180). Adequate dietary fiber supply is a major challenge for participants in high altitude expeditions due to limited access to fresh food outside base camp. In the studies conducted so far, mountaineers most often did not cover the dietary requirements for this component for adults i.e., the values did not exceed 25 g/day (57, 83, 84). Recommended dietary fiber supply in athletes is 38 g (71). Insufficient intake of dietary fiber can result in a deterioration of intestinal function, as well as a reduction in the diversity of the microbiota (181). Prebiotics (galacto-oligosaccharides, fructo-oligosaccharides, human milk oligosaccharides, xylo-oligosaccharides, mannano-oligosaccharides, inulin), which are one type of fiber, are substrates selectively utilized by host microorganisms (182, 183). Prebiotic supplementation in high-altitude conditions is worth considering in view of the low dietary fiber supply, and the well-documented effect of prebiotics in promoting SCFA production.

Similarly, probiotics, or live microorganisms, when consumed in adequate amounts, can provide health benefits (184). Long-term intake of probiotics *Bulgaricus 2038* and *Streptococcus thermophilus 1131* can increase the abundance of *Bacteroides* closely related to butyrate and propionate production (185). Increasing iron absorption may be a valuable strategy for improving iron status while at high altitudes and before going on an expedition. The probiotic strain *Lactobacillus plantarum 299v* (Lp299v, LP299V®) has been shown to improve iron absorption in meal studies (186, 187). The results of the study indicate that ingestion of Lp299v with 20 mg of iron may result in a more significant and faster improvement in iron levels compared to iron alone (102). The study was conducted in female athletes with low iron stores at sea level, so the effectiveness of the strategy would also need to be tested in high-altitude conditions. More studies using prebiotics and probiotics in high-altitude conditions are needed to assess their effectiveness in protecting the intestinal barrier against hypoxia-induced stress.

#### 3.5.7 Omega-3 fatty acids

Long-chain omega-3 polyunsaturated fatty acids, such as docosahexaenoic acid (DHA), eicosapentaenoic acid (EPA) and alpha-linolenic acid (ALA), show anti-inflammatory effects (188). According to the recommendations of the European Food Safety Agency (189), their intake by adults should be 250 mg for EPA and DHA and 0.5% of total energy intake for ALA. EPA/DHA are considered safe in doses of up to 5 g/day (190). The results of some studies suggest that EPA/DHA may improve endurance, delay the onset of muscle soreness and promote recovery in athletes (191, 192). It has also been shown that supplementation affects muscle protein synthesis, especially under conditions of immobilization and energy restriction or when taken with other nutrients (193, 194). A study using cell cultures assessed that DHA attenuates the increase in total ROS production and lipid peroxidation in mature adipocytes. The importance of such changes observed in response to DHA may reduce the deleterious effects of excessive ROS production caused by severe hypoxia and prevent lipid peroxidation and damage (195). In high-altitude conditions, the provision of adequate omega-3 fatty acids may be inadequate especially above the base camp due to limited access to foods abundant in omega-3 fatty acids. However, further human studies conducted under hypoxic conditions and at terrestrial altitude are needed to unequivocally recommend omega-3 fatty acid supplementation in high-altitude conditions.

#### 3.5.8 Curcumin

Polyphenol curcumin derived from the *Curcuma longa* plant provides protection of the intestinal barrier by reducing oxidative stress and apoptosis (196). In the situation of HAPE, curcumin supplementation increases the integrity of the alveolar epithelial barrier (197). Curcumin supplementation may lead to lower levels of pro-inflammatory cytokines or indirect markers of muscle damage under normoxia (198–200). However, more studies conducted under hypoxic conditions and at terrestrial altitude are needed to demonstrate the efficacy of curcumin supplementation to justify its use in mountaineers.

#### 3.5.9 Glutamine

Glutamine is an amino acid with potent anti-inflammatory effects, a key energy fuel for rapidly growing cells (such as immune cells and enterocytes), a precursor to glucose, proteins and nucleic acids (201). It has a variety of functions in the body, including being a key component for the synthesis of cytokines, hormones, maintenance of acid-base balance, ammonia transport and cell proliferation (202). Under hypoxic conditions, glutamine synthesis in the body may be insufficient to meet the increased demand for this amino acid. Findings indicate that glutamine supplementation reduces the risk of upper respiratory tract infections after prolonged low-altitude (1,640 m) exercise, thereby improving immunocompetence in hypoxia (203). We evaluated the effects of glutamine supplementation on the regulation of lymphocyte-mediated immune responses and inflammation in humans exposed to 6 h of simulated hypoxia at 4,500 m in a normobaric chamber, with and without exercise (204). Glutamine (at a dose of 20 g) was administered to study participants for 6 days before exposure to altitude. Glutamine supplementation stimulated both T helper 1 lymphocyte responses, inhibiting T helper 2 lymphocyte responses, and attenuated the release of IL-6 and TNF-α (204). Glutamine may have a positive effect on the function of enterocytes and immune cells in the gut and improve defense mechanisms. Moreover, it can be an energy substrate for the intestinal microbiota, reducing the inflammatory process accompanied by increased intestinal permeability, thereby improving cognitive function and mood (205, 206). Glutamine administered to rats at a dose of 5 g/kg of b.w. 3 days before and 5 days during a simulated 7,000 m altitude had the effect of reducing hypoxia-induced damage to the intestinal structure and regulating the intestinal microbiota (207). Glutamine can directly modify neurotransmitter activity in the brain, affecting mood and cognitive function (208). McMorris et al. showed in a meta-analysis that hypoxia harms cognitive processes (209), and this condition can probably be alleviated by glutamine supplementation (210), but there is a lack of human studies using glutamine supplementation in high-altitude conditions to confirm this hypothesis. Studies in hypoxia and at terrestrial altitude, with and without glutamine supplementation, should be performed to experimentally correlate the relationship between glutamine and cognitive function, mood, immune response in hypoxia.

#### 3.5.10 N-acetylcysteine (NAC)

N-acetylcysteine (NAC) is used for its antioxidant and anti-inflammatory effects. NAC supplementation appears to be safe and may regulate glutathione homeostasis, have antioxidant effects and improve physical performance (211). In studies conducted to date, NAC has increased resistance to fatigue (212), improved immune function (213), as well as hemodynamics and muscle blood flow (214) and modulated EPO production (215). The dosage of NAC in the conducted studies ranged from 600 to 1,200 mg per day, and supplementation was applied for 5–9 days (214, 215). There is a lack of studies confirming the validity of NAC supplementation in hypoxic conditions. Moreover, there are also studies available that do not confirm a positive effect of NAC on EPO production (216) and even indicate a pro-oxidant effect when taken in high doses before a prolonged period of time (i.e., 1,200 mg/day for 4 weeks, then 2,400 mg/day for another 2 weeks) (217). Thus, further research is needed to evaluate NAC as a useful supplement for use before or during altitude exposure.

#### 3.5.11 Tart cherry juice

Supplementation with tart cherry taken in many forms can improve muscle recovery from exercise-induced damage (218–220), physical performance (221, 222) and sleep quality (223–225.) It is recommended to use tart cherry juice at a dose of about 250–350 ml (30 ml for concentrate), twice a day, for 4–5 days before sports competition or for 2–3 days afterwards to support recovery (141). The results of a study conducted by Horiuchi et al. (226) indicate that a 5-day supplementation with tart cherry (a capsule containing 100 mg of anthocyanins) improves exercise tolerance in hypoxia. However, more studies are needed to unequivocally recommend supplementation with this preparation in hypoxia.

New arrivals to altitude commonly experience poor-quality sleep. At high altitudes, reduced oxygen content in the blood can cause sleep disturbances with frequent awakenings and feelings of shortness of breath (227). De Aquino Lemos et al. (228) have found that hypoxia reduces total sleep time, sleep efficiency and causes rapid eye movements. Changes in sleep patterns can affect mood and cognitive function after 24 h (228). In mountaineers rapidly climbing to high altitudes, sleep quality is initially impaired, but improves with acclimatization due to improved oxygen saturation (229). Given the positive effects of tart cherry juice on sleep duration and efficiency, the use of this supplement in high-altitude conditions seems an interesting strategy. Further studies need to be conducted in hypoxia and at terrestrial altitude, with and without tart cherry juice supplementation, to experimentally correlate the relationship between tart cherry juice and physical performance and sleep quality in hypoxia.

#### 3.5.12 Ginko biloba

*Ginko biloba* herb extract has been found to reduce tissue hypoxia, induce vasodilation, and reduce free radical generation and lung leakage, which in turn may prevent AMS (230). A meta-analysis conducted by Tsai et al. yielded inconclusive results regarding the effect of *Ginko biloba* on AMS prevention (230). In the study, Moraga et al. showed that 24 h pretreatment with *Ginko biloba* and subsequent maintenance during high-altitude exposure was sufficient to reduce the incidence of AMS in participants with no prior high-altitude experience (231).

### 3.6 Practical tips for nutrition at different stages of a high altitude expedition

Staying at high altitudes in the mountains requires advance planning of nutrition, hydration and supplementation. Depending on the direction of activities, expeditions vary in the location of the base camp and its supplies, so it is necessary to have knowledge of the expedition's logistics. Mountaineers can procure food on their own or use the help of an expedition agency, which organizes kitchen facilities at base camp and provides food, and is responsible for obtaining drinking water (139). Much of the food that will be consumed during the expedition is obtained in the country where the mountain activities are undertaken. Cultural differences, religious aspects and the availability of products in a country should be taken into account. In Buddhist countries, such as Nepal, it can be difficult to purchase meat products.

Depending on the stage of the expedition, mountaineers face some difficulties regarding nutrition and hydration. The first is trekking and acclimatization. This stage usually ends with an approach to base camp at an altitude of about 3,500–4,500 meters above sea level, depending on the mountain range. Mountaineers during this time usually enjoy overnight accommodations in high mountain villages. Eating in local restaurants, tasting street food and drinking water can cause food poisoning and contagious diarrhea. Therefore, extreme caution should be applied and care should be taken to ensure the hygiene of the meals consumed.

At base camp (for peaks >5,000 m), mountaineers are usually located at an altitude between 4,500 and 5,000 meters above sea level. This is the place where mountain climbers can enjoy a well-balanced diet, laden with fresh foods for the last time before setting off on a summit attack. Expeditions without the help of mountain travel agencies and hired porters are forced to properly plan the distribution of transported food. Food supplies should secure the mountaineers’ stay at altitude for several weeks, which requires an appropriate weight and volume strategy, such as low-weight high-energy foods and freeze-dried products, to transport the maximum amount of high-quality and tasty food. Trying to avoid diet monotony is hard work, but it helps motivate mountaineers to eat. Storage of fruits and vegetables is a problem due to low temperatures. Animal products, such as eggs and meat (poultry and yak meat), are good sources of protein during the expedition, but their availability is limited. Another source of protein is fish, usually in canned form. In Buddhist countries, base camps usually use vegetable protein from pulses such as lentils and chickpeas. To maintain nutritional hygiene, latrines should be placed away from kitchens and water sources.

Above the base camp, mountaineers use glacial water for hydration and for cooking. At this stage, mountain climbers move to the next point, the intermediate base camp, gaining more and more altitude on their way to the summit. Mountaineers also descend to these points after gaining altitude to acclimatize above base camp for better recovery and sleep. It is essential to properly plan and pack freeze-dried and dehydrated foods, carbohydrate snacks and supplements to ensure an adequate supply of energy on the way to the summit. Reduced appetite, low temperatures and the problem of cooking meals and water at high altitude in an unfavorable environment pose major challenges for mountaineers, which increase with the altitude gained. Regardless of appetite, it is necessary to eat regular meals and high-energy snacks throughout the day.

During a summit attack there is an accumulation of unfavorable factors. Fatigue and weakness increase, aerobic capacity decreases due to staying at very high or extreme altitudes (often in the death zone i.e., >8,000 m), and in addition, mountaineers are exposed to very low temperatures, the consequence of which is sometimes frostbite (232). Throughout the year, the typical chill equivalent temperature near the summit of Everest is always <−30°C, and the typical facial frostbite time is less than 20 min (232). Bad weather is estimated to contribute to 25% of deaths in the mountains (233). The summit attack, including the return to intermediate camp, may take 12–24 h (139), and this time depends on the mountain range and the logistics of this stage. The food taken by mountaineers must be at this stage as light as possible, high-energy, and also easily digestible in order not to burden the weakened digestive system. Drinks and food taken should be properly protected from freezing, preferably by keeping them close to the body in the inner pockets of the suit. It is worth considering energy gels, gummies, carbohydrate jellies and water with added carbohydrates i.e., high-energy, high-carbohydrate, low-weight products and supplements. Due to the described difficulties of staying in high mountains and climbing, it is important to increase athletes’ awareness of nutrition, hydration and supplementation in order to improve body function, general wellbeing, exercise performance and increase their chances of success.

## 4 Summary and further directions

Due to the negative effects of weather and environmental conditions in high mountains, mountaineers are at risk of compromised health and even loss of life. Adequate preparation for an expedition, especially attention to factors that mountaineers have control over, including nutrition, hydration and supplementation, can help prevent the development of nutritional deficiencies that affect the deterioration of health and performance. Energy requirements during climbing in high-altitude conditions can vary significantly depending on the load in the form of equipment transported in the backpack, the experience of the climber, as well as the difficulties encountered during the climb. Mountaineers, while at high-extreme altitudes, are at risk of energy deficiency, and consequently loss of body weight and lean body mass. Despite the lack of appetite, care should be taken to ensure regularity in the meals eaten and snacks taken.

The supply of carbohydrates in the diet of mountaineers should be adapted to the stage of the expedition and the intensity of the effort. Staying at base camp, with low physical activity, this supply should be about 3-5 g CHO per kg of b.w./day, while during trekking and/or climbing—a minimum of 6 g/kg of b.w./day. In view of the endurance physical effort mountaineers undertake, dietary protein supply should be a minimum of 1.4 g/kg of b.w./day, taking into account high-quality protein and distributing it to main meals and snacks during climbing and ascent to the summit. Mountaineers should rely on valuable sources of fat, including in their diet products rich in poly- and monounsaturated fatty acids such as vegetable oils (olive oil, flaxseed oil), nuts, seeds. It is recommended that the supply of fat in the diet of mountaineers should be in addition to energy after the carbohydrate and protein requirements are met, and should be 20%–35% of the total energy supply. Mountaineers choosing products in the mountain diet should be guided primarily by appetite and adjust the supply of this component to the individual tolerance of the digestive system.

Limited access to fresh fruits and vegetables and protein-containing foods in high-mountain conditions may be associated with an inadequate supply of minerals and vitamins. Ferritin and vitamin D serum concentration should be assessed before the expedition. If there is a deficiency of micronutrients, such as iron and vitamin D, supplementation should be implemented to optimize health and continued under supervision of a medical doctor. It is worth including foods rich in iron in the diet and ensuring adequate iron bioavailability. It is advisable to adjust the appropriate dose of vitamin D supplementation to the current serum concentration and continue supplementation during the expedition. Mountaineers should consume products rich in antioxidants, including sources of vitamin C, beta-carotene, vitamin E, selenium and zinc while at high altitude. Freeze-dried fruits, vegetables and their powdered forms can be valuable sources of antioxidant vitamins in high-altitude conditions. Further research is needed to formulate recommendations for the supply of micronutrients in high altitude conditions.

Fluid supply at high altitude should be individualized due to different rates of fluid and electrolyte loss, urine color control (dehydration results in a darker urine colour) and symptoms of AMS, which causes water retention in the body. A regular supply of fluids is necessary to prevent dehydration.

Due to poor access to food products at high and extreme altitudes, supplementation may be the only solution to provide adequate energy and essential nutrients, as well as to improve the functioning of the body in harsh environmental conditions. The potential ergogenic effect of supplemented carbohydrates on exercise capacity in high-mountain conditions may be modulated by acclimatization. More research is needed to develop appropriate carbohydrate supply strategies that take into account the effects of hypoxia on insulin sensitivity and substrate oxidation to optimize the activities performed in high-altitude conditions. It is worth considering supplementation with a protein supplement to cover the daily requirement of this ingredient due to the lower availability of food products that are sources of protein in high-altitude conditions, especially above base camp. There is still a lack of studies evaluating the effect of protein supplementation on feelings of satiety at high altitude. Pro-oxidant-antioxidant balance is paramount for altitude acclimatization, but the question of planning an appropriate antioxidant-related nutritional and supplementation strategy remains unclear.

Caffeine administered in low doses (3 mg/kg of b.w.) has the potential to positively affect mountaineers’ cognitive function. Preliminary data suggest that caffeine may be a potential treatment for high altitude pulmonary edema. However, further studies are needed to assess the efficacy of caffeine supplementation in hypoxic conditions. Nitrates supplemented in the form of beet juice may improve endothelial function at high altitude, but nitrates are not recommended as a prophylactic or ergogenic agent for already existing altitude disease. Further studies are needed to evaluate the validity of beet juice supplementation in high-altitude conditions. In view of the gastrointestinal complaints reported during high altitude climbing, probiotics and prebiotics have the potential to protect mountaineers’ intestinal barrier from hypoxia-induced dysfunction. However, further research focusing on the use of specific probiotic strains and their effects on the state of the gut microbiota in high-mountain conditions is needed. In high-altitude conditions, the provision of adequate omega-3 fatty acids may be inadequate especially above base camp due to limited access to foods abundant in omega-3 fatty acids. Further research is needed to assess the validity of omega-3 supplementation in hypoxic conditions. Glutamine may have a positive effect on the function of enterocytes and immune cells in the gut. Moreover, it may be an energy substrate for the intestinal microbiota, reducing the inflammatory process accompanied by increased intestinal permeability, thereby improving cognitive function and mood. Further research is needed with humans using glutamine supplementation in high-altitude conditions. There are several supplements, such as curcumin, *Ginko biloba*, NAC and tart cherry juice, that need further research to evaluate their effectiveness in high-altitude conditions. Given the positive effect of tart cherry juice on sleep quality and physical performance, the use of this supplement in high-altitude conditions seems an interesting strategy. There is a lack of well-designed studies examining the relationship between the use of dietary supplements and improved athletic performance and health at high-extreme altitudes. Therefore, there is a need for controlled, randomized studies involving mountaineers.

## 5 Conclusions

In view of the difficulties of being in high mountains and practicing alpine climbing, as described in the review, it is important to increase athletes’ awareness of nutrition, hydration and supplementation in order to improve well-being, physical performance and increase the chance of success in high altitude action, and to provide the appropriate dietary care necessary to educate mountaineers and personalize recommendations to the needs of the individual.
